# Supplementary material for: Transcriptome Analysis Reveals the Stress Tolerance to and Accumulation Mechanisms of Cadmium in Paspalum vaginatum Swartz
Source: Plants (Basel). 2022 Aug 9;11(16):2078. doi: 10.3390/plants11162078 (PMC9414793; doi:10.3390/plants11162078)
Supplement: Supplementary file 1 [file plants-11-02078-s001.zip › plants-1769036-supplementary.pdf]

Table S1. Graphs present 17 KEGG pathways with the highest transcriptional variations, out of the down-regulated DEGs in *Paspalum vaginatum*

Term Name	MainClass	GeneHitsInSelectedSet	p-value	enrichFactor	GeneListInSelectedSets
00280 Valine, leucine and isoleucine degradation	A09100 Metabolism	28	5.86E-07	2.806163	TRINITY_DN127_c0_g2_i1_6, TRINITY_DN37349_c0_g1_i1_6, TRINITY_DN35781_c0_g1_i1_4, TRINITY_DN5569_c0_g2_i1_4, TRINITY_DN32766_c0_g1_i1_4, TRINITY_DN31880_c0_g1_i1_4, TRINITY_DN36297_c0_g1_i1_4, TRINITY_DN30746_c0_g1_i1_4, TRINITY_DN36089_c0_g1_i1_4, TRINITY_DN29435_c0_g1_i1_6, TRINITY_DN37543_c0_g1_i1_6, TRINITY_DN27651_c0_g1_i1_6, TRINITY_DN36033_c0_g1_i1_4, TRINITY_DN37346_c0_g1_i1_4, TRINITY_DN27451_c0_g1_i1_4, TRINITY_DN38780_c0_g1_i1_6, TRINITY_DN31588_c0_g1_i1_4, TRINITY_DN28649_c0_g1_i1_4, TRINITY_DN26605_c0_g1_i1_4, TRINITY_DN31675_c0_g1_i1_6, TRINITY_DN7306_c0_g1_i1_4, TRINITY_DN5158_c0_g2_i1_4, TRINITY_DN33593_c0_g1_i1_4, TRINITY_DN34083_c0_g1_i1_4, TRINITY_DN4679_c0_g1_i1_6, TRINITY_DN26364_c0_g1_i1_4, TRINITY_DN34962_c0_g1_i1_4, TRINITY_DN34394_c0_g1_i1_4

03400 DNA repair and recombination proteins	A09180 Brite Hierarchies	175	1.46E-05	1.263987	TRINITY_DN30255_c0_g1_i1_4, TRINITY_DN25720_c0_g2_i1_4, TRINITY_DN32182_c0_g1_i1_4, TRINITY_DN26817_c0_g1_i1_4, TRINITY_DN35285_c0_g1_i1_4, TRINITY_DN36276_c0_g1_i1_4, TRINITY_DN14215_c0_g1_i1_4, TRINITY_DN12061_c0_g1_i1_4, TRINITY_DN35364_c0_g1_i1_4, TRINITY_DN20376_c0_g3_i1_4, TRINITY_DN16863_c0_g1_i1_4, TRINITY_DN27369_c0_g1_i1_4, TRINITY_DN25759_c0_g1_i2_6, TRINITY_DN5471_c0_g1_i1_6, TRINITY_DN13697_c0_g1_i1_4, TRINITY_DN34538_c0_g1_i1_4, TRINITY_DN34330_c0_g1_i1_4, TRINITY_DN27594_c0_g1_i1_3, TRINITY_DN30179_c0_g1_i1_6, TRINITY_DN13485_c0_g2_i1_6, TRINITY_DN13667_c0_g2_i1_6, TRINITY_DN28906_c0_g1_i1_4, TRINITY_DN27637_c0_g1_i1_4, TRINITY_DN36002_c0_g1_i1_4, TRINITY_DN30434_c0_g1_i1_4, TRINITY_DN32780_c0_g1_i1_6, TRINITY_DN3051_c0_g1_i2_4, TRINITY_DN38907_c0_g1_i1_6, TRINITY_DN16241_c0_g1_i1_4, TRINITY_DN16771_c0_g1_i1_4, TRINITY_DN36191_c0_g1_i1_5, TRINITY_DN31332_c0_g1_i1_4, TRINITY_DN28886_c0_g1_i1_4, TRINITY_DN35014_c0_g1_i1_4, TRINITY_DN19169_c0_g1_i1_4, TRINITY_DN26722_c0_g1_i1_4, TRINITY_DN37118_c0_g1_i1_4, TRIN-

	ITY_DN35334_c0_g1_i1_6, TRIN-<br>ITY_DN32522_c0_g1_i1_4, TRIN-<br>ITY_DN8776_c0_g1_i1_4, TRIN-<br>ITY_DN16779_c0_g1_i1_4, TRIN-<br>ITY_DN30595_c0_g1_i1_6, TRIN-<br>ITY_DN33144_c0_g1_i1_6, TRIN-<br>ITY_DN36814_c0_g1_i1_4, TRIN-<br>ITY_DN725_c0_g1_i2_3, TRIN-<br>ITY_DN34934_c0_g1_i1_4, TRIN-<br>ITY_DN31793_c0_g1_i1_4, TRIN-<br>ITY_DN17127_c0_g1_i1_4, TRIN-<br>ITY_DN23064_c0_g1_i1_4, TRIN-<br>ITY_DN6610_c0_g1_i1_4, TRIN-<br>ITY_DN16329_c0_g2_i1_6, TRIN-<br>ITY_DN8590_c0_g1_i1_4, TRIN-<br>ITY_DN10031_c0_g1_i1_4, TRIN-<br>ITY_DN1829_c0_g1_i1_5, TRIN-<br>ITY_DN27649_c0_g1_i1_4, TRIN-<br>ITY_DN33746_c0_g1_i1_6, TRIN-<br>ITY_DN29455_c0_g1_i1_4, TRIN-<br>ITY_DN34648_c0_g1_i1_4, TRIN-<br>ITY_DN37452_c0_g1_i1_6, TRIN-<br>ITY_DN33306_c0_g1_i1_4, TRIN-<br>ITY_DN33907_c0_g1_i1_6, TRIN-<br>ITY_DN4470_c0_g1_i1_4, TRIN-<br>ITY_DN38092_c0_g1_i1_6, TRIN-<br>ITY_DN34868_c0_g1_i1_4, TRIN-<br>ITY_DN33713_c0_g1_i1_6, TRIN-<br>ITY_DN19233_c0_g1_i2_4, TRIN-<br>ITY_DN29340_c0_g1_i1_6, TRIN-<br>ITY_DN5155_c0_g1_i1_6, TRIN-<br>ITY_DN28722_c0_g1_i1_6, TRIN-<br>ITY_DN34741_c0_g1_i1_4, TRIN-<br>ITY_DN30011_c0_g1_i1_4, TRIN-<br>ITY_DN33944_c0_g1_i1_4, TRIN-<br>ITY_DN19175_c0_g1_i1_4, TRIN-<br>ITY_DN37243_c0_g1_i1_4, TRIN-

	ITY_DN27534_c0_g1_i1_4, TRIN-ITY_DN11525_c0_g1_i1_4, TRIN-ITY_DN25144_c1_g2_i3_4, TRIN-ITY_DN618_c0_g1_i1_4, TRIN-ITY_DN32394_c0_g1_i1_4, TRIN-ITY_DN31327_c0_g1_i1_6, TRIN-ITY_DN27161_c4_g1_i1_6, TRIN-ITY_DN25554_c1_g3_i4_4, TRIN-ITY_DN12830_c0_g1_i2_6, TRIN-ITY_DN35711_c0_g1_i1_4, TRIN-ITY_DN9067_c0_g1_i1_4, TRIN-ITY_DN5787_c0_g1_i1_4, TRIN-ITY_DN8486_c0_g1_i1_4, TRIN-ITY_DN34113_c0_g1_i1_4, TRIN-ITY_DN6589_c0_g1_i1_4, TRIN-ITY_DN31030_c0_g1_i1_4, TRIN-ITY_DN30875_c0_g1_i1_4, TRIN-ITY_DN27258_c0_g1_i1_3, TRIN-ITY_DN35991_c0_g1_i1_4, TRIN-ITY_DN1129_c0_g1_i1_6, TRIN-ITY_DN10754_c0_g1_i1_4, TRIN-ITY_DN2047_c0_g1_i2_2, TRIN-ITY_DN35716_c0_g1_i1_4, TRIN-ITY_DN26770_c0_g1_i1_4, TRIN-ITY_DN27536_c0_g1_i1_4, TRIN-ITY_DN32243_c0_g1_i1_4, TRIN-ITY_DN28951_c0_g1_i1_5, TRIN-ITY_DN37299_c0_g1_i1_4, TRIN-ITY_DN30300_c0_g1_i1_4, TRIN-ITY_DN2191_c0_g1_i1_4, TRIN-ITY_DN36754_c0_g1_i1_4, TRIN-ITY_DN5025_c0_g1_i2_2, TRIN-ITY_DN19706_c0_g1_i1_4, TRIN-ITY_DN13304_c0_g1_i2_4, TRIN-ITY_DN30604_c0_g1_i1_6, TRIN-ITY_DN30753_c0_g1_i1_4, TRIN-ITY_DN6328_c0_g1_i1_6, TRIN-

	ITY_DN9983_c0_g1_i1_4, TRIN-<br>ITY_DN3255_c0_g1_i1_4, TRIN-<br>ITY_DN31202_c0_g1_i1_6, TRIN-<br>ITY_DN27566_c0_g1_i1_4, TRIN-<br>ITY_DN11018_c0_g1_i1_4, TRIN-<br>ITY_DN26008_c0_g1_i1_6, TRIN-<br>ITY_DN36231_c0_g1_i1_4, TRIN-<br>ITY_DN37276_c0_g1_i1_6, TRIN-<br>ITY_DN33065_c0_g1_i1_6, TRIN-<br>ITY_DN17840_c0_g1_i1_4, TRIN-<br>ITY_DN27218_c0_g1_i1_4, TRIN-<br>ITY_DN26111_c0_g1_i1_4, TRIN-<br>ITY_DN27811_c0_g1_i1_6, TRIN-<br>ITY_DN15806_c0_g2_i1_4, TRIN-<br>ITY_DN6891_c0_g1_i1_6, TRIN-<br>ITY_DN6149_c0_g1_i1_6, TRIN-<br>ITY_DN30254_c0_g1_i1_4, TRIN-<br>ITY_DN16768_c0_g1_i1_4, TRIN-<br>ITY_DN13494_c0_g1_i1_6, TRIN-<br>ITY_DN18056_c0_g1_i1_4, TRIN-<br>ITY_DN10336_c0_g2_i1_4, TRIN-<br>ITY_DN3908_c0_g1_i1_4, TRIN-<br>ITY_DN28034_c0_g1_i1_3, TRIN-<br>ITY_DN14813_c0_g1_i1_4, TRIN-<br>ITY_DN32804_c0_g1_i1_4, TRIN-<br>ITY_DN5688_c0_g1_i1_4, TRIN-<br>ITY_DN36955_c0_g1_i1_6, TRIN-<br>ITY_DN7388_c0_g1_i1_6, TRIN-<br>ITY_DN28052_c0_g1_i1_4, TRIN-<br>ITY_DN36641_c0_g1_i1_4, TRIN-<br>ITY_DN32834_c0_g1_i1_4, TRIN-<br>ITY_DN16313_c0_g1_i1_4, TRIN-<br>ITY_DN34137_c0_g1_i1_4, TRIN-<br>ITY_DN30352_c0_g1_i1_6, TRIN-<br>ITY_DN34997_c0_g1_i1_6, TRIN-<br>ITY_DN12403_c0_g1_i1_4, TRIN-<br>ITY_DN29811_c0_g1_i1_6, TRIN-

					ITY_DN28652_c0_g1_i1_4, TRIN-ITY_DN30904_c0_g1_i1_4, TRIN-ITY_DN14947_c0_g1_i1_4, TRIN-ITY_DN35240_c0_g1_i1_4, TRIN-ITY_DN31839_c0_g1_i1_6, TRIN-ITY_DN18488_c0_g1_i1_4, TRIN-ITY_DN35586_c0_g1_i1_4, TRIN-ITY_DN12617_c0_g1_i2_4, TRIN-ITY_DN10675_c0_g1_i1_4, TRIN-ITY_DN36306_c0_g1_i1_4, TRIN-ITY_DN346_c0_g1_i1_4, TRIN-ITY_DN30110_c0_g1_i1_4, TRIN-ITY_DN29690_c0_g1_i1_2, TRIN-ITY_DN35718_c0_g1_i1_4, TRIN-ITY_DN34291_c0_g1_i1_4, TRIN-ITY_DN14747_c0_g1_i1_6, TRIN-ITY_DN9318_c0_g1_i1_4, TRIN-ITY_DN29568_c0_g1_i1_4, TRIN-ITY_DN25759_c0_g2_i1_6, TRIN-ITY_DN20949_c0_g1_i1_4, TRIN-ITY_DN10198_c0_g2_i1_4, TRIN-ITY_DN4058_c0_g1_i1_6, TRIN-ITY_DN36727_c0_g1_i1_4, TRIN-ITY_DN28569_c0_g1_i1_6, TRIN-ITY_DN14709_c0_g1_i1_5, TRIN-ITY_DN2936_c0_g1_i1_4, TRIN-ITY_DN36766_c0_g1_i1_4
A09120 Genetic Information Processing	A09120 Genetic Information Processing	193	1.51E-04	1.19562	TRINITY_DN30255_c0_g1_i1_4, TRINITY_DN32182_c0_g1_i1_4, TRINITY_DN26817_c0_g1_i1_4, TRINITY_DN31947_c0_g1_i1_4, TRINITY_DN35285_c0_g1_i1_4, TRINITY_DN36276_c0_g1_i1_4, TRINITY_DN12061_c0_g1_i1_4, TRINITY_DN35364_c0_g1_i1_4, TRINITY_DN34538_c0_g1_i1_4, TRINITY_DN27594_c0_g1_i1_3,

	TRINITY_DN13667_c0_g2_i1_6, TRINITY_DN36002_c0_g1_i1_4, TRINITY_DN30434_c0_g1_i1_4, TRINITY_DN16771_c0_g1_i1_4, TRINITY_DN36191_c0_g1_i1_5, TRINITY_DN19169_c0_g1_i1_4, TRINITY_DN26722_c0_g1_i1_4, TRINITY_DN37118_c0_g1_i1_4, TRINITY_DN35334_c0_g1_i1_6, TRINITY_DN8776_c0_g1_i1_4, TRINITY_DN33144_c0_g1_i1_6, TRINITY_DN34934_c0_g1_i1_4, TRINITY_DN31793_c0_g1_i1_4, TRINITY_DN17127_c0_g1_i1_4, TRINITY_DN23064_c0_g1_i1_4, TRINITY_DN8590_c0_g1_i1_4, TRINITY_DN1829_c0_g1_i1_5, TRINITY_DN27649_c0_g1_i1_4, TRINITY_DN33746_c0_g1_i1_6, TRINITY_DN34648_c0_g1_i1_4, TRINITY_DN33313_c0_g1_i1_4, TRINITY_DN5155_c0_g1_i1_6, TRINITY_DN34741_c0_g1_i1_4, TRINITY_DN30011_c0_g1_i1_4, TRINITY_DN33944_c0_g1_i1_4, TRINITY_DN27534_c0_g1_i1_4, TRINITY_DN618_c0_g1_i1_4, TRINITY_DN31327_c0_g1_i1_6, TRINITY_DN12830_c0_g1_i2_6, TRINITY_DN35711_c0_g1_i1_4, TRINITY_DN9067_c0_g1_i1_4, TRINITY_DN5787_c0_g1_i1_4, TRINITY_DN8486_c0_g1_i1_4, TRINITY_DN31030_c0_g1_i1_4, TRINITY_DN27258_c0_g1_i1_3, TRINITY_DN1129_c0_g1_i1_6, TRINITY_DN2047_c0_g1_i2_2, TRIN-

	ITY_DN13304_c0_g1_i2_4, TRIN-
	ITY_DN30604_c0_g1_i1_6, TRIN-
	ITY_DN30604_c0_g1_i1_4, TRIN-
	ITY_DN31202_c0_g1_i1_6, TRIN-
	ITY_DN27566_c0_g1_i1_4, TRIN-
	ITY_DN26008_c0_g1_i1_6, TRIN-
	ITY_DN36231_c0_g1_i1_4, TRIN-
	ITY_DN37276_c0_g1_i1_6, TRIN-
	ITY_DN33065_c0_g1_i1_6, TRIN-
	ITY_DN17840_c0_g1_i1_4, TRIN-
	ITY_DN27218_c0_g1_i1_4, TRIN-
	ITY_DN27811_c0_g1_i1_6, TRIN-
	ITY_DN6891_c0_g1_i1_6, TRIN-
	ITY_DN18056_c0_g1_i1_4, TRIN-
	ITY_DN10336_c0_g2_i1_4, TRIN-
	ITY_DN29389_c0_g1_i1_4, TRIN-
	ITY_DN14813_c0_g1_i1_4, TRIN-
	ITY_DN32834_c0_g1_i1_4, TRIN-
	ITY_DN30352_c0_g1_i1_6, TRIN-
	ITY_DN8568_c0_g1_i1_4, TRIN-
	ITY_DN29811_c0_g1_i1_6, TRIN-
	ITY_DN28652_c0_g1_i1_4, TRIN-
	ITY_DN30904_c0_g1_i1_4, TRIN-
	ITY_DN36395_c0_g1_i1_4, TRIN-
	ITY_DN35240_c0_g1_i1_4, TRIN-
	ITY_DN36306_c0_g1_i1_4, TRIN-
	ITY_DN29690_c0_g1_i1_2, TRIN-
	ITY_DN26835_c0_g1_i1_4, TRIN-
	ITY_DN34291_c0_g1_i1_4, TRIN-
	ITY_DN14747_c0_g1_i1_6, TRIN-
	ITY_DN9318_c0_g1_i1_4, TRIN-
	ITY_DN29568_c0_g1_i1_4, TRIN-
	ITY_DN20949_c0_g1_i1_4, TRIN-
	ITY_DN26616_c0_g1_i1_4, TRIN-
	ITY_DN14709_c0_g1_i1_5, TRIN-
	ITY_DN2936_c0_g1_i1_4, TRIN-
	ITY_DN36766_c0_g1_i1_4, TRIN-

	ITY_DN25720_c0_g2_i1_4, TRIN-<br>ITY_DN28880_c0_g1_i1_4, TRIN-<br>ITY_DN20918_c0_g1_i1_4, TRIN-<br>ITY_DN14215_c0_g1_i1_4, TRIN-<br>ITY_DN20376_c0_g3_i1_4, TRIN-<br>ITY_DN16863_c0_g1_i1_4, TRIN-<br>ITY_DN27369_c0_g1_i1_4, TRIN-<br>ITY_DN25759_c0_g1_i2_6, TRIN-<br>ITY_DN5471_c0_g1_i1_6, TRIN-<br>ITY_DN13697_c0_g1_i1_4, TRIN-<br>ITY_DN34330_c0_g1_i1_4, TRIN-<br>ITY_DN30179_c0_g1_i1_6, TRIN-<br>ITY_DN13485_c0_g2_i1_6, TRIN-<br>ITY_DN28906_c0_g1_i1_4, TRIN-<br>ITY_DN27637_c0_g1_i1_4, TRIN-<br>ITY_DN32780_c0_g1_i1_6, TRIN-<br>ITY_DN3051_c0_g1_i2_4, TRIN-<br>ITY_DN38907_c0_g1_i1_6, TRIN-<br>ITY_DN16241_c0_g1_i1_4, TRIN-<br>ITY_DN28926_c0_g1_i1_4, TRIN-<br>ITY_DN31332_c0_g1_i1_4, TRIN-<br>ITY_DN28886_c0_g1_i1_4, TRIN-<br>ITY_DN35014_c0_g1_i1_4, TRIN-<br>ITY_DN29693_c0_g1_i1_4, TRIN-<br>ITY_DN32522_c0_g1_i1_4, TRIN-<br>ITY_DN16779_c0_g1_i1_4, TRIN-<br>ITY_DN30595_c0_g1_i1_6, TRIN-<br>ITY_DN36814_c0_g1_i1_4, TRIN-<br>ITY_DN725_c0_g1_i2_3, TRIN-<br>ITY_DN6610_c0_g1_i1_4, TRIN-<br>ITY_DN16329_c0_g2_i1_6, TRIN-<br>ITY_DN10031_c0_g1_i1_4, TRIN-<br>ITY_DN29455_c0_g1_i1_4, TRIN-<br>ITY_DN37452_c0_g1_i1_6, TRIN-<br>ITY_DN33306_c0_g1_i1_4, TRIN-<br>ITY_DN33907_c0_g1_i1_6, TRIN-<br>ITY_DN4470_c0_g1_i1_4, TRIN-

ITY_DN38092_c0_g1_i1_6, TRIN-
ITY_DN34868_c0_g1_i1_4, TRIN-
ITY_DN33713_c0_g1_i1_6, TRIN-
ITY_DN19233_c0_g1_i2_4, TRIN-
ITY_DN34048_c0_g1_i1_4, TRIN-
ITY_DN29340_c0_g1_i1_6, TRIN-
ITY_DN28722_c0_g1_i1_6, TRIN-
ITY_DN29940_c0_g1_i1_4, TRIN-
ITY_DN34287_c0_g1_i1_4, TRIN-
ITY_DN19175_c0_g1_i1_4, TRIN-
ITY_DN37243_c0_g1_i1_4, TRIN-
ITY_DN11525_c0_g1_i1_4, TRIN-
ITY_DN25144_c1_g2_i3_4, TRIN-
ITY_DN32394_c0_g1_i1_4, TRIN-
ITY_DN27161_c4_g1_i1_6, TRIN-
ITY_DN25554_c1_g3_i4_4, TRIN-
ITY_DN34113_c0_g1_i1_4, TRIN-
ITY_DN6589_c0_g1_i1_4, TRIN-
ITY_DN30875_c0_g1_i1_4, TRIN-
ITY_DN35991_c0_g1_i1_4, TRIN-
ITY_DN29512_c0_g1_i1_4, TRIN-
ITY_DN10754_c0_g1_i1_4, TRIN-
ITY_DN35716_c0_g1_i1_4, TRIN-
ITY_DN26770_c0_g1_i1_4, TRIN-
ITY_DN27536_c0_g1_i1_4, TRIN-
ITY_DN32243_c0_g1_i1_4, TRIN-
ITY_DN28951_c0_g1_i1_5, TRIN-
ITY_DN37299_c0_g1_i1_4, TRIN-
ITY_DN30300_c0_g1_i1_4, TRIN-
ITY_DN2191_c0_g1_i1_4, TRIN-
ITY_DN36754_c0_g1_i1_4, TRIN-
ITY_DN5025_c0_g1_i2_2, TRIN-
ITY_DN19706_c0_g1_i1_4, TRIN-
ITY_DN30753_c0_g1_i1_4, TRIN-
ITY_DN6328_c0_g1_i1_6, TRIN-
ITY_DN9983_c0_g1_i1_4, TRIN-
ITY_DN3255_c0_g1_i1_4, TRIN-

	ITY_DN26251_c0_g1_i1_4, TRINITY_DN11018_c0_g1_i1_4, TRINITY_DN26111_c0_g1_i1_4, TRINITY_DN15806_c0_g2_i1_4, TRINITY_DN6149_c0_g1_i1_6, TRINITY_DN30254_c0_g1_i1_4, TRINITY_DN16768_c0_g1_i1_4, TRINITY_DN13494_c0_g1_i1_6, TRINITY_DN3908_c0_g1_i1_4, TRINITY_DN28034_c0_g1_i1_3, TRINITY_DN32804_c0_g1_i1_4, TRINITY_DN5688_c0_g1_i1_4, TRINITY_DN33073_c0_g1_i1_4, TRINITY_DN36955_c0_g1_i1_6, TRINITY_DN7388_c0_g1_i1_6, TRINITY_DN28052_c0_g1_i1_4, TRINITY_DN36641_c0_g1_i1_4, TRINITY_DN16313_c0_g1_i1_4, TRINITY_DN34137_c0_g1_i1_4, TRINITY_DN34997_c0_g1_i1_6, TRINITY_DN12403_c0_g1_i1_4, TRINITY_DN14947_c0_g1_i1_4, TRINITY_DN31839_c0_g1_i1_6, TRINITY_DN18488_c0_g1_i1_4, TRINITY_DN35586_c0_g1_i1_4, TRINITY_DN12617_c0_g1_i2_4, TRINITY_DN10675_c0_g1_i1_4, TRINITY_DN346_c0_g1_i1_4, TRINITY_DN30110_c0_g1_i1_4, TRINITY_DN35718_c0_g1_i1_4, TRINITY_DN25759_c0_g2_i1_6, TRINITY_DN10198_c0_g2_i1_4, TRINITY_DN4058_c0_g1_i1_6, TRINITY_DN36727_c0_g1_i1_4, TRINITY_DN28569_c0_g1_i1_6

00564 Glycerophos-pholipid metabolism	A09100 Metabo-lism	25	5.87E-04	2.020566377	TRINITY_DN31687_c0_g1_i1_4, TRINITY_DN127_c0_g2_i1_6, TRIN-ITY_DN3062_c0_g2_i1_4, TRIN-ITY_DN31880_c0_g1_i1_4, TRIN-ITY_DN33884_c0_g1_i1_4, TRIN-ITY_DN30746_c0_g1_i1_4, TRIN-ITY_DN29435_c0_g1_i1_6, TRIN-ITY_DN30161_c0_g1_i1_6, TRIN-ITY_DN3293_c0_g1_i1_4, TRIN-ITY_DN37346_c0_g1_i1_4, TRIN-ITY_DN27451_c0_g1_i1_4, TRIN-ITY_DN27394_c0_g1_i1_4, TRIN-ITY_DN38780_c0_g1_i1_6, TRIN-ITY_DN31588_c0_g1_i1_4, TRIN-ITY_DN35640_c0_g1_i1_4, TRIN-ITY_DN28649_c0_g1_i1_4, TRIN-ITY_DN26605_c0_g1_i1_4, TRIN-ITY_DN31949_c0_g1_i1_4, TRIN-ITY_DN29834_c0_g1_i1_6, TRIN-ITY_DN29924_c0_g1_i1_4, TRIN-ITY_DN35824_c0_g1_i1_4, TRIN-ITY_DN33593_c0_g1_i1_4, TRIN-ITY_DN4679_c0_g1_i1_6, TRIN-ITY_DN27308_c0_g1_i1_4, TRIN-ITY_DN37350_c0_g1_i1_4
03021 Transcription machinery	A09180 Brite Hier-archies	182	8.10E-04	1.179652998	TRINITY_DN30255_c0_g1_i1_4, TRINITY_DN25720_c0_g2_i1_4, TRINITY_DN32182_c0_g1_i1_4, TRINITY_DN26817_c0_g1_i1_4, TRINITY_DN35285_c0_g1_i1_4, TRINITY_DN36276_c0_g1_i1_4, TRINITY_DN20918_c0_g1_i1_4, TRINITY_DN14215_c0_g1_i1_4, TRINITY_DN12061_c0_g1_i1_4, TRINITY_DN35364_c0_g1_i1_4, TRINITY_DN20376_c0_g3_i1_4, TRINITY_DN16863_c0_g1_i1_4,

TRINITY_DN27369_c0_g1_i1_4, TRINITY_DN25759_c0_g1_i2_6, TRINITY_DN5471_c0_g1_i1_6, TRINITY_DN13697_c0_g1_i1_4, TRINITY_DN34538_c0_g1_i1_4, TRINITY_DN34330_c0_g1_i1_4, TRINITY_DN27594_c0_g1_i1_3, TRINITY_DN30179_c0_g1_i1_6, TRINITY_DN13485_c0_g2_i1_6, TRINITY_DN13667_c0_g2_i1_6, TRINITY_DN28906_c0_g1_i1_4, TRINITY_DN27637_c0_g1_i1_4, TRINITY_DN36002_c0_g1_i1_4, TRINITY_DN30434_c0_g1_i1_4, TRINITY_DN32780_c0_g1_i1_6, TRINITY_DN3051_c0_g1_i2_4, TRINITY_DN38907_c0_g1_i1_6, TRINITY_DN16241_c0_g1_i1_4, TRINITY_DN16771_c0_g1_i1_4, TRINITY_DN36191_c0_g1_i1_5, TRINITY_DN31332_c0_g1_i1_4, TRINITY_DN28886_c0_g1_i1_4, TRINITY_DN35014_c0_g1_i1_4, TRINITY_DN19169_c0_g1_i1_4, TRINITY_DN26722_c0_g1_i1_4, TRINITY_DN37118_c0_g1_i1_4, TRINITY_DN35334_c0_g1_i1_6, TRINITY_DN32522_c0_g1_i1_4, TRINITY_DN8776_c0_g1_i1_4, TRINITY_DN16779_c0_g1_i1_4, TRINITY_DN30595_c0_g1_i1_6, TRINITY_DN33144_c0_g1_i1_6, TRINITY_DN36814_c0_g1_i1_4, TRINITY_DN725_c0_g1_i2_3, TRINITY_DN34934_c0_g1_i1_4, TRINITY_DN31793_c0_g1_i1_4, TRINITY_DN17127_c0_g1_i1_4, TRIN-

ITY_DN23064_c0_g1_i1_4, TRIN-<br>ITY_DN6610_c0_g1_i1_4, TRIN-<br>ITY_DN16329_c0_g2_i1_6, TRIN-<br>ITY_DN8590_c0_g1_i1_4, TRIN-<br>ITY_DN10031_c0_g1_i1_4, TRIN-<br>ITY_DN1829_c0_g1_i1_5, TRIN-<br>ITY_DN27649_c0_g1_i1_4, TRIN-<br>ITY_DN33746_c0_g1_i1_6, TRIN-<br>ITY_DN29455_c0_g1_i1_4, TRIN-<br>ITY_DN34648_c0_g1_i1_4, TRIN-<br>ITY_DN37452_c0_g1_i1_6, TRIN-<br>ITY_DN33306_c0_g1_i1_4, TRIN-<br>ITY_DN33907_c0_g1_i1_6, TRIN-<br>ITY_DN4470_c0_g1_i1_4, TRIN-<br>ITY_DN38092_c0_g1_i1_6, TRIN-<br>ITY_DN34868_c0_g1_i1_4, TRIN-<br>ITY_DN33713_c0_g1_i1_6, TRIN-<br>ITY_DN19233_c0_g1_i2_4, TRIN-<br>ITY_DN34048_c0_g1_i1_4, TRIN-<br>ITY_DN29340_c0_g1_i1_6, TRIN-<br>ITY_DN5155_c0_g1_i1_6, TRIN-<br>ITY_DN28722_c0_g1_i1_6, TRIN-<br>ITY_DN34741_c0_g1_i1_4, TRIN-<br>ITY_DN34287_c0_g1_i1_4, TRIN-<br>ITY_DN30011_c0_g1_i1_4, TRIN-<br>ITY_DN33944_c0_g1_i1_4, TRIN-<br>ITY_DN19175_c0_g1_i1_4, TRIN-<br>ITY_DN37243_c0_g1_i1_4, TRIN-<br>ITY_DN27534_c0_g1_i1_4, TRIN-<br>ITY_DN11525_c0_g1_i1_4, TRIN-<br>ITY_DN25144_c1_g2_i3_4, TRIN-<br>ITY_DN618_c0_g1_i1_4, TRIN-<br>ITY_DN32394_c0_g1_i1_4, TRIN-<br>ITY_DN31327_c0_g1_i1_6, TRIN-<br>ITY_DN27161_c4_g1_i1_6, TRIN-<br>ITY_DN25554_c1_g3_i4_4, TRIN-<br>ITY_DN12830_c0_g1_i2_6, TRIN-

	ITY_DN35711_c0_g1_i1_4, TRINITY_DN9067_c0_g1_i1_4, TRINITY_DN5787_c0_g1_i1_4, TRINITY_DN8486_c0_g1_i1_4, TRINITY_DN34113_c0_g1_i1_4, TRINITY_DN6589_c0_g1_i1_4, TRINITY_DN31030_c0_g1_i1_4, TRINITY_DN30875_c0_g1_i1_4, TRINITY_DN27258_c0_g1_i1_3, TRINITY_DN35991_c0_g1_i1_4, TRINITY_DN1129_c0_g1_i1_6, TRINITY_DN10754_c0_g1_i1_4, TRINITY_DN2047_c0_g1_i2_2, TRINITY_DN35716_c0_g1_i1_4, TRINITY_DN26770_c0_g1_i1_4, TRINITY_DN27536_c0_g1_i1_4, TRINITY_DN32243_c0_g1_i1_4, TRINITY_DN28951_c0_g1_i1_5, TRINITY_DN37299_c0_g1_i1_4, TRINITY_DN30300_c0_g1_i1_4, TRINITY_DN2191_c0_g1_i1_4, TRINITY_DN36754_c0_g1_i1_4, TRINITY_DN5025_c0_g1_i2_2, TRINITY_DN19706_c0_g1_i1_4, TRINITY_DN13304_c0_g1_i2_4, TRINITY_DN30604_c0_g1_i1_6, TRINITY_DN30753_c0_g1_i1_4, TRINITY_DN30604_c0_g1_i1_4, TRINITY_DN6328_c0_g1_i1_6, TRINITY_DN9983_c0_g1_i1_4, TRINITY_DN3255_c0_g1_i1_4, TRINITY_DN26251_c0_g1_i1_4, TRINITY_DN31202_c0_g1_i1_6, TRINITY_DN27566_c0_g1_i1_4, TRINITY_DN11018_c0_g1_i1_4, TRINITY_DN26008_c0_g1_i1_6, TRINITY_DN36231_c0_g1_i1_4, TRIN-

ITY_DN37276_c0_g1_i1_6, TRIN-
ITY_DN33065_c0_g1_i1_6, TRIN-
ITY_DN17840_c0_g1_i1_4, TRIN-
ITY_DN27218_c0_g1_i1_4, TRIN-
ITY_DN26111_c0_g1_i1_4, TRIN-
ITY_DN27811_c0_g1_i1_6, TRIN-
ITY_DN15806_c0_g2_i1_4, TRIN-
ITY_DN6891_c0_g1_i1_6, TRIN-
ITY_DN6149_c0_g1_i1_6, TRIN-
ITY_DN30254_c0_g1_i1_4, TRIN-
ITY_DN16768_c0_g1_i1_4, TRIN-
ITY_DN13494_c0_g1_i1_6, TRIN-
ITY_DN18056_c0_g1_i1_4, TRIN-
ITY_DN10336_c0_g2_i1_4, TRIN-
ITY_DN29389_c0_g1_i1_4, TRIN-
ITY_DN3908_c0_g1_i1_4, TRIN-
ITY_DN28034_c0_g1_i1_3, TRIN-
ITY_DN14813_c0_g1_i1_4, TRIN-
ITY_DN32804_c0_g1_i1_4, TRIN-
ITY_DN5688_c0_g1_i1_4, TRIN-
ITY_DN33073_c0_g1_i1_4, TRIN-
ITY_DN36955_c0_g1_i1_6, TRIN-
ITY_DN7388_c0_g1_i1_6, TRIN-
ITY_DN28052_c0_g1_i1_4, TRIN-
ITY_DN36641_c0_g1_i1_4, TRIN-
ITY_DN32834_c0_g1_i1_4, TRIN-
ITY_DN16313_c0_g1_i1_4, TRIN-
ITY_DN34137_c0_g1_i1_4, TRIN-
ITY_DN30352_c0_g1_i1_6, TRIN-
ITY_DN34997_c0_g1_i1_6, TRIN-
ITY_DN12403_c0_g1_i1_4, TRIN-
ITY_DN29811_c0_g1_i1_6, TRIN-
ITY_DN28652_c0_g1_i1_4, TRIN-
ITY_DN30904_c0_g1_i1_4, TRIN-
ITY_DN14947_c0_g1_i1_4, TRIN-
ITY_DN35240_c0_g1_i1_4, TRIN-
ITY_DN31839_c0_g1_i1_6, TRIN-

						ITY_DN18488_c0_g1_i1_4, TRIN-ITY_DN35586_c0_g1_i1_4, TRIN-ITY_DN12617_c0_g1_i2_4, TRIN-ITY_DN10675_c0_g1_i1_4, TRIN-ITY_DN36306_c0_g1_i1_4, TRIN-ITY_DN346_c0_g1_i1_4, TRIN-ITY_DN30110_c0_g1_i1_4, TRIN-ITY_DN29690_c0_g1_i1_2, TRIN-ITY_DN35718_c0_g1_i1_4, TRIN-ITY_DN34291_c0_g1_i1_4, TRIN-ITY_DN14747_c0_g1_i1_6, TRIN-ITY_DN9318_c0_g1_i1_4, TRIN-ITY_DN29568_c0_g1_i1_4, TRIN-ITY_DN25759_c0_g2_i1_6, TRIN-ITY_DN20949_c0_g1_i1_4, TRIN-ITY_DN10198_c0_g2_i1_4, TRIN-ITY_DN4058_c0_g1_i1_6, TRIN-ITY_DN36727_c0_g1_i1_4, TRIN-ITY_DN28569_c0_g1_i1_6, TRIN-ITY_DN14709_c0_g1_i1_5, TRIN-ITY_DN2936_c0_g1_i1_4, TRIN-ITY_DN36766_c0_g1_i1_4
B	09121 Transcrip-tion	A09120 Genetic Information Pro-cessing	182	8.10E-04	1.179652998	TRINITY_DN30255_c0_g1_i1_4, TRINITY_DN25720_c0_g2_i1_4, TRINITY_DN32182_c0_g1_i1_4, TRINITY_DN26817_c0_g1_i1_4, TRINITY_DN35285_c0_g1_i1_4, TRINITY_DN36276_c0_g1_i1_4, TRINITY_DN20918_c0_g1_i1_4, TRINITY_DN14215_c0_g1_i1_4, TRINITY_DN12061_c0_g1_i1_4, TRINITY_DN35364_c0_g1_i1_4, TRINITY_DN20376_c0_g3_i1_4, TRINITY_DN16863_c0_g1_i1_4, TRINITY_DN27369_c0_g1_i1_4, TRINITY_DN25759_c0_g1_i2_6,

	TRINITY_DN5471_c0_g1_i1_6, TRINITY_DN13697_c0_g1_i1_4, TRINITY_DN34538_c0_g1_i1_4, TRINITY_DN34330_c0_g1_i1_4, TRINITY_DN27594_c0_g1_i1_3, TRINITY_DN30179_c0_g1_i1_6, TRINITY_DN13485_c0_g2_i1_6, TRINITY_DN13667_c0_g2_i1_6, TRINITY_DN28906_c0_g1_i1_4, TRINITY_DN27637_c0_g1_i1_4, TRINITY_DN36002_c0_g1_i1_4, TRINITY_DN30434_c0_g1_i1_4, TRINITY_DN32780_c0_g1_i1_6, TRINITY_DN3051_c0_g1_i2_4, TRINITY_DN38907_c0_g1_i1_6, TRINITY_DN16241_c0_g1_i1_4, TRINITY_DN16771_c0_g1_i1_4, TRINITY_DN36191_c0_g1_i1_5, TRINITY_DN31332_c0_g1_i1_4, TRINITY_DN28886_c0_g1_i1_4, TRINITY_DN35014_c0_g1_i1_4, TRINITY_DN19169_c0_g1_i1_4, TRINITY_DN26722_c0_g1_i1_4, TRINITY_DN37118_c0_g1_i1_4, TRINITY_DN35334_c0_g1_i1_6, TRINITY_DN32522_c0_g1_i1_4, TRINITY_DN8776_c0_g1_i1_4, TRINITY_DN16779_c0_g1_i1_4, TRINITY_DN30595_c0_g1_i1_6, TRINITY_DN33144_c0_g1_i1_6, TRINITY_DN36814_c0_g1_i1_4, TRINITY_DN725_c0_g1_i2_3, TRINITY_DN34934_c0_g1_i1_4, TRINITY_DN31793_c0_g1_i1_4, TRINITY_DN17127_c0_g1_i1_4, TRINITY_DN23064_c0_g1_i1_4, TRINITY_DN6610_c0_g1_i1_4, TRIN-

ITY_DN16329_c0_g2_i1_6, TRIN-
ITY_DN8590_c0_g1_i1_4, TRIN-
ITY_DN10031_c0_g1_i1_4, TRIN-
ITY_DN1829_c0_g1_i1_5, TRIN-
ITY_DN27649_c0_g1_i1_4, TRIN-
ITY_DN33746_c0_g1_i1_6, TRIN-
ITY_DN29455_c0_g1_i1_4, TRIN-
ITY_DN34648_c0_g1_i1_4, TRIN-
ITY_DN37452_c0_g1_i1_6, TRIN-
ITY_DN33306_c0_g1_i1_4, TRIN-
ITY_DN33907_c0_g1_i1_6, TRIN-
ITY_DN4470_c0_g1_i1_4, TRIN-
ITY_DN38092_c0_g1_i1_6, TRIN-
ITY_DN34868_c0_g1_i1_4, TRIN-
ITY_DN33713_c0_g1_i1_6, TRIN-
ITY_DN19233_c0_g1_i2_4, TRIN-
ITY_DN34048_c0_g1_i1_4, TRIN-
ITY_DN29340_c0_g1_i1_6, TRIN-
ITY_DN5155_c0_g1_i1_6, TRIN-
ITY_DN28722_c0_g1_i1_6, TRIN-
ITY_DN34741_c0_g1_i1_4, TRIN-
ITY_DN34287_c0_g1_i1_4, TRIN-
ITY_DN30011_c0_g1_i1_4, TRIN-
ITY_DN33944_c0_g1_i1_4, TRIN-
ITY_DN19175_c0_g1_i1_4, TRIN-
ITY_DN37243_c0_g1_i1_4, TRIN-
ITY_DN27534_c0_g1_i1_4, TRIN-
ITY_DN11525_c0_g1_i1_4, TRIN-
ITY_DN25144_c1_g2_i3_4, TRIN-
ITY_DN618_c0_g1_i1_4, TRIN-
ITY_DN32394_c0_g1_i1_4, TRIN-
ITY_DN31327_c0_g1_i1_6, TRIN-
ITY_DN27161_c4_g1_i1_6, TRIN-
ITY_DN25554_c1_g3_i4_4, TRIN-
ITY_DN12830_c0_g1_i2_6, TRIN-
ITY_DN35711_c0_g1_i1_4, TRIN-
ITY_DN9067_c0_g1_i1_4, TRIN-

		ITY_DN5787_c0_g1_i1_4, TRIN-<br>ITY_DN8486_c0_g1_i1_4, TRIN-<br>ITY_DN34113_c0_g1_i1_4, TRIN-<br>ITY_DN6589_c0_g1_i1_4, TRIN-<br>ITY_DN31030_c0_g1_i1_4, TRIN-<br>ITY_DN30875_c0_g1_i1_4, TRIN-<br>ITY_DN27258_c0_g1_i1_3, TRIN-<br>ITY_DN35991_c0_g1_i1_4, TRIN-<br>ITY_DN1129_c0_g1_i1_6, TRIN-<br>ITY_DN10754_c0_g1_i1_4, TRIN-<br>ITY_DN2047_c0_g1_i2_2, TRIN-<br>ITY_DN35716_c0_g1_i1_4, TRIN-<br>ITY_DN26770_c0_g1_i1_4, TRIN-<br>ITY_DN27536_c0_g1_i1_4, TRIN-<br>ITY_DN32243_c0_g1_i1_4, TRIN-<br>ITY_DN28951_c0_g1_i1_5, TRIN-<br>ITY_DN37299_c0_g1_i1_4, TRIN-<br>ITY_DN30300_c0_g1_i1_4, TRIN-<br>ITY_DN2191_c0_g1_i1_4, TRIN-<br>ITY_DN36754_c0_g1_i1_4, TRIN-<br>ITY_DN5025_c0_g1_i2_2, TRIN-<br>ITY_DN19706_c0_g1_i1_4, TRIN-<br>ITY_DN13304_c0_g1_i2_4, TRIN-<br>ITY_DN30604_c0_g1_i1_6, TRIN-<br>ITY_DN30753_c0_g1_i1_4, TRIN-<br>ITY_DN30604_c0_g1_i1_4, TRIN-<br>ITY_DN6328_c0_g1_i1_6, TRIN-<br>ITY_DN9983_c0_g1_i1_4, TRIN-<br>ITY_DN3255_c0_g1_i1_4, TRIN-<br>ITY_DN26251_c0_g1_i1_4, TRIN-<br>ITY_DN31202_c0_g1_i1_6, TRIN-<br>ITY_DN27566_c0_g1_i1_4, TRIN-<br>ITY_DN11018_c0_g1_i1_4, TRIN-<br>ITY_DN26008_c0_g1_i1_6, TRIN-<br>ITY_DN36231_c0_g1_i1_4, TRIN-<br>ITY_DN37276_c0_g1_i1_6, TRIN-<br>ITY_DN33065_c0_g1_i1_6, TRIN-

ITY_DN17840_c0_g1_i1_4, TRIN- ITY_DN27218_c0_g1_i1_4, TRIN- ITY_DN26111_c0_g1_i1_4, TRIN- ITY_DN27811_c0_g1_i1_6, TRIN- ITY_DN15806_c0_g2_i1_4, TRIN- ITY_DN6891_c0_g1_i1_6, TRIN- ITY_DN6149_c0_g1_i1_6, TRIN- ITY_DN30254_c0_g1_i1_4, TRIN- ITY_DN16768_c0_g1_i1_4, TRIN- ITY_DN13494_c0_g1_i1_6, TRIN- ITY_DN18056_c0_g1_i1_4, TRIN- ITY_DN10336_c0_g2_i1_4, TRIN- ITY_DN29389_c0_g1_i1_4, TRIN- ITY_DN3908_c0_g1_i1_4, TRIN- ITY_DN28034_c0_g1_i1_3, TRIN- ITY_DN14813_c0_g1_i1_4, TRIN- ITY_DN32804_c0_g1_i1_4, TRIN- ITY_DN5688_c0_g1_i1_4, TRIN- ITY_DN33073_c0_g1_i1_4, TRIN- ITY_DN36955_c0_g1_i1_6, TRIN- ITY_DN7388_c0_g1_i1_6, TRIN- ITY_DN28052_c0_g1_i1_4, TRIN- ITY_DN36641_c0_g1_i1_4, TRIN- ITY_DN32834_c0_g1_i1_4, TRIN- ITY_DN16313_c0_g1_i1_4, TRIN- ITY_DN34137_c0_g1_i1_4, TRIN- ITY_DN30352_c0_g1_i1_6, TRIN- ITY_DN34997_c0_g1_i1_6, TRIN- ITY_DN12403_c0_g1_i1_4, TRIN- ITY_DN29811_c0_g1_i1_6, TRIN- ITY_DN28652_c0_g1_i1_4, TRIN- ITY_DN30904_c0_g1_i1_4, TRIN- ITY_DN14947_c0_g1_i1_4, TRIN- ITY_DN35240_c0_g1_i1_4, TRIN- ITY_DN31839_c0_g1_i1_6, TRIN- ITY_DN18488_c0_g1_i1_4, TRIN- ITY_DN35586_c0_g1_i1_4, TRIN-

					ITY_DN12617_c0_g1_i2_4, TRINITY_DN10675_c0_g1_i1_4, TRINITY_DN36306_c0_g1_i1_4, TRINITY_DN346_c0_g1_i1_4, TRINITY_DN30110_c0_g1_i1_4, TRINITY_DN29690_c0_g1_i1_2, TRINITY_DN35718_c0_g1_i1_4, TRINITY_DN34291_c0_g1_i1_4, TRINITY_DN14747_c0_g1_i1_6, TRINITY_DN9318_c0_g1_i1_4, TRINITY_DN29568_c0_g1_i1_4, TRINITY_DN25759_c0_g2_i1_6, TRINITY_DN20949_c0_g1_i1_4, TRINITY_DN10198_c0_g2_i1_4, TRINITY_DN4058_c0_g1_i1_6, TRINITY_DN36727_c0_g1_i1_4, TRINITY_DN28569_c0_g1_i1_6, TRINITY_DN14709_c0_g1_i1_5, TRINITY_DN2936_c0_g1_i1_4, TRINITY_DN36766_c0_g1_i1_4
03020 RNA polymerase	A09120 Genetic Information Processing	182	8.10E-04	1.179652998	TRINITY_DN30255_c0_g1_i1_4, TRINITY_DN25720_c0_g2_i1_4, TRINITY_DN32182_c0_g1_i1_4, TRINITY_DN26817_c0_g1_i1_4, TRINITY_DN35285_c0_g1_i1_4, TRINITY_DN36276_c0_g1_i1_4, TRINITY_DN20918_c0_g1_i1_4, TRINITY_DN14215_c0_g1_i1_4, TRINITY_DN12061_c0_g1_i1_4, TRINITY_DN35364_c0_g1_i1_4, TRINITY_DN20376_c0_g3_i1_4, TRINITY_DN16863_c0_g1_i1_4, TRINITY_DN27369_c0_g1_i1_4, TRINITY_DN25759_c0_g1_i2_6, TRINITY_DN5471_c0_g1_i1_6, TRINITY_DN13697_c0_g1_i1_4, TRIN-

	ITY_DN34538_c0_g1_i1_4, TRIN-ITY_DN34330_c0_g1_i1_4, TRIN-ITY_DN27594_c0_g1_i1_3, TRIN-ITY_DN30179_c0_g1_i1_6, TRIN-ITY_DN13485_c0_g2_i1_6, TRIN-ITY_DN13667_c0_g2_i1_6, TRIN-ITY_DN28906_c0_g1_i1_4, TRIN-ITY_DN27637_c0_g1_i1_4, TRIN-ITY_DN36002_c0_g1_i1_4, TRIN-ITY_DN30434_c0_g1_i1_4, TRIN-ITY_DN32780_c0_g1_i1_6, TRIN-ITY_DN3051_c0_g1_i2_4, TRIN-ITY_DN38907_c0_g1_i1_6, TRIN-ITY_DN16241_c0_g1_i1_4, TRIN-ITY_DN16771_c0_g1_i1_4, TRIN-ITY_DN36191_c0_g1_i1_5, TRIN-ITY_DN31332_c0_g1_i1_4, TRIN-ITY_DN28886_c0_g1_i1_4, TRIN-ITY_DN35014_c0_g1_i1_4, TRIN-ITY_DN19169_c0_g1_i1_4, TRIN-ITY_DN26722_c0_g1_i1_4, TRIN-ITY_DN37118_c0_g1_i1_4, TRIN-ITY_DN35334_c0_g1_i1_6, TRIN-ITY_DN32522_c0_g1_i1_4, TRIN-ITY_DN8776_c0_g1_i1_4, TRIN-ITY_DN16779_c0_g1_i1_4, TRIN-ITY_DN30595_c0_g1_i1_6, TRIN-ITY_DN33144_c0_g1_i1_6, TRIN-ITY_DN36814_c0_g1_i1_4, TRIN-ITY_DN725_c0_g1_i2_3, TRIN-ITY_DN34934_c0_g1_i1_4, TRIN-ITY_DN31793_c0_g1_i1_4, TRIN-ITY_DN17127_c0_g1_i1_4, TRIN-ITY_DN23064_c0_g1_i1_4, TRIN-ITY_DN6610_c0_g1_i1_4, TRIN-ITY_DN16329_c0_g2_i1_6, TRIN-ITY_DN8590_c0_g1_i1_4, TRIN-

	ITY_DN10031_c0_g1_i1_4, TRIN-ITY_DN1829_c0_g1_i1_5, TRIN-ITY_DN27649_c0_g1_i1_4, TRIN-ITY_DN33746_c0_g1_i1_6, TRIN-ITY_DN29455_c0_g1_i1_4, TRIN-ITY_DN34648_c0_g1_i1_4, TRIN-ITY_DN37452_c0_g1_i1_6, TRIN-ITY_DN33306_c0_g1_i1_4, TRIN-ITY_DN33907_c0_g1_i1_6, TRIN-ITY_DN4470_c0_g1_i1_4, TRIN-ITY_DN38092_c0_g1_i1_6, TRIN-ITY_DN34868_c0_g1_i1_4, TRIN-ITY_DN33713_c0_g1_i1_6, TRIN-ITY_DN19233_c0_g1_i2_4, TRIN-ITY_DN34048_c0_g1_i1_4, TRIN-ITY_DN29340_c0_g1_i1_6, TRIN-ITY_DN5155_c0_g1_i1_6, TRIN-ITY_DN28722_c0_g1_i1_6, TRIN-ITY_DN34741_c0_g1_i1_4, TRIN-ITY_DN34287_c0_g1_i1_4, TRIN-ITY_DN30011_c0_g1_i1_4, TRIN-ITY_DN33944_c0_g1_i1_4, TRIN-ITY_DN19175_c0_g1_i1_4, TRIN-ITY_DN37243_c0_g1_i1_4, TRIN-ITY_DN27534_c0_g1_i1_4, TRIN-ITY_DN11525_c0_g1_i1_4, TRIN-ITY_DN25144_c1_g2_i3_4, TRIN-ITY_DN618_c0_g1_i1_4, TRIN-ITY_DN32394_c0_g1_i1_4, TRIN-ITY_DN31327_c0_g1_i1_6, TRIN-ITY_DN27161_c4_g1_i1_6, TRIN-ITY_DN25554_c1_g3_i4_4, TRIN-ITY_DN12830_c0_g1_i2_6, TRIN-ITY_DN35711_c0_g1_i1_4, TRIN-ITY_DN9067_c0_g1_i1_4, TRIN-ITY_DN5787_c0_g1_i1_4, TRIN-ITY_DN8486_c0_g1_i1_4, TRIN-

		ITY_DN34113_c0_g1_i1_4, TRIN-<br>ITY_DN6589_c0_g1_i1_4, TRIN-<br>ITY_DN31030_c0_g1_i1_4, TRIN-<br>ITY_DN30875_c0_g1_i1_4, TRIN-<br>ITY_DN27258_c0_g1_i1_3, TRIN-<br>ITY_DN35991_c0_g1_i1_4, TRIN-<br>ITY_DN1129_c0_g1_i1_6, TRIN-<br>ITY_DN10754_c0_g1_i1_4, TRIN-<br>ITY_DN2047_c0_g1_i2_2, TRIN-<br>ITY_DN35716_c0_g1_i1_4, TRIN-<br>ITY_DN26770_c0_g1_i1_4, TRIN-<br>ITY_DN27536_c0_g1_i1_4, TRIN-<br>ITY_DN32243_c0_g1_i1_4, TRIN-<br>ITY_DN28951_c0_g1_i1_5, TRIN-<br>ITY_DN37299_c0_g1_i1_4, TRIN-<br>ITY_DN30300_c0_g1_i1_4, TRIN-<br>ITY_DN2191_c0_g1_i1_4, TRIN-<br>ITY_DN36754_c0_g1_i1_4, TRIN-<br>ITY_DN5025_c0_g1_i2_2, TRIN-<br>ITY_DN19706_c0_g1_i1_4, TRIN-<br>ITY_DN13304_c0_g1_i2_4, TRIN-<br>ITY_DN30604_c0_g1_i1_6, TRIN-<br>ITY_DN30753_c0_g1_i1_4, TRIN-<br>ITY_DN30604_c0_g1_i1_4, TRIN-<br>ITY_DN6328_c0_g1_i1_6, TRIN-<br>ITY_DN9983_c0_g1_i1_4, TRIN-<br>ITY_DN3255_c0_g1_i1_4, TRIN-<br>ITY_DN26251_c0_g1_i1_4, TRIN-<br>ITY_DN31202_c0_g1_i1_6, TRIN-<br>ITY_DN27566_c0_g1_i1_4, TRIN-<br>ITY_DN11018_c0_g1_i1_4, TRIN-<br>ITY_DN26008_c0_g1_i1_6, TRIN-<br>ITY_DN36231_c0_g1_i1_4, TRIN-<br>ITY_DN37276_c0_g1_i1_6, TRIN-<br>ITY_DN33065_c0_g1_i1_6, TRIN-<br>ITY_DN17840_c0_g1_i1_4, TRIN-<br>ITY_DN27218_c0_g1_i1_4, TRIN-

		ITY_DN26111_c0_g1_i1_4, TRIN-ITY_DN27811_c0_g1_i1_6, TRIN-ITY_DN15806_c0_g2_i1_4, TRIN-ITY_DN6891_c0_g1_i1_6, TRIN-ITY_DN6149_c0_g1_i1_6, TRIN-ITY_DN30254_c0_g1_i1_4, TRIN-ITY_DN16768_c0_g1_i1_4, TRIN-ITY_DN13494_c0_g1_i1_6, TRIN-ITY_DN18056_c0_g1_i1_4, TRIN-ITY_DN10336_c0_g2_i1_4, TRIN-ITY_DN29389_c0_g1_i1_4, TRIN-ITY_DN3908_c0_g1_i1_4, TRIN-ITY_DN28034_c0_g1_i1_3, TRIN-ITY_DN14813_c0_g1_i1_4, TRIN-ITY_DN32804_c0_g1_i1_4, TRIN-ITY_DN5688_c0_g1_i1_4, TRIN-ITY_DN33073_c0_g1_i1_4, TRIN-ITY_DN36955_c0_g1_i1_6, TRIN-ITY_DN7388_c0_g1_i1_6, TRIN-ITY_DN28052_c0_g1_i1_4, TRIN-ITY_DN36641_c0_g1_i1_4, TRIN-ITY_DN32834_c0_g1_i1_4, TRIN-ITY_DN16313_c0_g1_i1_4, TRIN-ITY_DN34137_c0_g1_i1_4, TRIN-ITY_DN30352_c0_g1_i1_6, TRIN-ITY_DN34997_c0_g1_i1_6, TRIN-ITY_DN12403_c0_g1_i1_4, TRIN-ITY_DN29811_c0_g1_i1_6, TRIN-ITY_DN28652_c0_g1_i1_4, TRIN-ITY_DN30904_c0_g1_i1_4, TRIN-ITY_DN14947_c0_g1_i1_4, TRIN-ITY_DN35240_c0_g1_i1_4, TRIN-ITY_DN31839_c0_g1_i1_6, TRIN-ITY_DN18488_c0_g1_i1_4, TRIN-ITY_DN35586_c0_g1_i1_4, TRIN-ITY_DN12617_c0_g1_i2_4, TRIN-ITY_DN10675_c0_g1_i1_4, TRIN-

					ITY_DN36306_c0_g1_i1_4, TRINITY_DN346_c0_g1_i1_4, TRINITY_DN30110_c0_g1_i1_4, TRINITY_DN29690_c0_g1_i1_2, TRINITY_DN35718_c0_g1_i1_4, TRINITY_DN34291_c0_g1_i1_4, TRINITY_DN14747_c0_g1_i1_6, TRINITY_DN9318_c0_g1_i1_4, TRINITY_DN29568_c0_g1_i1_4, TRINITY_DN25759_c0_g2_i1_6, TRINITY_DN20949_c0_g1_i1_4, TRINITY_DN10198_c0_g2_i1_4, TRINITY_DN4058_c0_g1_i1_6, TRINITY_DN36727_c0_g1_i1_4, TRINITY_DN28569_c0_g1_i1_6, TRINITY_DN14709_c0_g1_i1_5, TRINITY_DN2936_c0_g1_i1_4, TRINITY_DN36766_c0_g1_i1_4
00561 Glycerolipid metabolism	A09100 Metabolism	20	0.002781088	1.96939046	TRINITY_DN35640_c0_g1_i1_4, TRINITY_DN31687_c0_g1_i1_4, TRINITY_DN127_c0_g2_i1_6, TRINITY_DN28649_c0_g1_i1_4, TRINITY_DN26605_c0_g1_i1_4, TRINITY_DN3062_c0_g2_i1_4, TRINITY_DN31880_c0_g1_i1_4, TRINITY_DN35824_c0_g1_i1_4, TRINITY_DN30746_c0_g1_i1_4, TRINITY_DN33593_c0_g1_i1_4, TRINITY_DN29435_c0_g1_i1_6, TRINITY_DN30161_c0_g1_i1_6, TRINITY_DN3293_c0_g1_i1_4, TRINITY_DN37346_c0_g1_i1_4, TRINITY_DN27451_c0_g1_i1_4, TRINITY_DN4679_c0_g1_i1_6, TRINITY_DN27394_c0_g1_i1_4, TRIN-

					ITY_DN38780_c0_g1_i1_6, TRIN-ITY_DN31588_c0_g1_i1_4, TRIN-ITY_DN27308_c0_g1_i1_4
04070 Phosphatidylin-ositol signaling sys-tem	A09130 Environ-mental Infor-mation Processing	2	0.00570907 7	15.03301384	TRINITY_DN35640_c0_g1_i1_4, TRINITY_DN27308_c0_g1_i1_4
00562 Inositol phos-phate metabolism	A09100 Metabo-lism	29	0.00757148 3	1.602784564	TRINITY_DN28340_c0_g1_i1_4, TRINITY_DN35675_c0_g1_i1_4, TRINITY_DN35781_c0_g1_i1_4, TRINITY_DN29389_c0_g1_i1_4, TRINITY_DN32766_c0_g1_i1_4, TRINITY_DN31108_c0_g1_i1_4, TRINITY_DN27558_c0_g1_i1_4, TRINITY_DN8825_c0_g2_i1_4, TRIN-ITY_DN33073_c0_g1_i1_4, TRIN-ITY_DN29435_c0_g1_i1_6, TRIN-ITY_DN29733_c0_g1_i1_4, TRIN-ITY_DN27651_c0_g1_i1_6, TRIN-ITY_DN35022_c0_g1_i1_4, TRIN-ITY_DN36253_c0_g1_i1_4, TRIN-ITY_DN32951_c0_g1_i1_6, TRIN-ITY_DN26955_c0_g1_i1_4, TRIN-ITY_DN35347_c0_g1_i1_4, TRIN-ITY_DN34048_c0_g1_i1_4, TRIN-ITY_DN33937_c0_g1_i1_4, TRIN-ITY_DN5158_c0_g2_i1_4, TRIN-ITY_DN33662_c0_g1_i1_6, TRIN-ITY_DN28916_c0_g1_i1_4, TRIN-ITY_DN28568_c0_g1_i1_4, TRIN-ITY_DN24015_c0_g1_i1_6, TRIN-ITY_DN31537_c0_g1_i1_4, TRIN-ITY_DN37684_c0_g1_i1_4, TRIN-ITY_DN36782_c0_g1_i1_4, TRIN-ITY_DN34962_c0_g1_i1_4, TRIN-ITY_DN30138_c0_g1_i1_4

B 09123 Folding, sorting and degradation	A09120 Genetic Information Processing	9	0.01083547 1	2.445128758	TRINITY_DN8568_c0_g1_i1_4, TRINITY_DN33313_c0_g1_i1_4, TRINITY_DN28880_c0_g1_i1_4, TRINITY_DN36395_c0_g1_i1_4, TRINITY_DN29693_c0_g1_i1_4, TRINITY_DN31947_c0_g1_i1_4, TRINITY_DN29512_c0_g1_i1_4, TRINITY_DN26616_c0_g1_i1_4, TRINITY_DN26835_c0_g1_i1_4
01002 Peptidases and inhibitors	A09180 Brite Hierarchies	9	0.01083547 1	2.445128758	TRINITY_DN8568_c0_g1_i1_4, TRINITY_DN33313_c0_g1_i1_4, TRINITY_DN28880_c0_g1_i1_4, TRINITY_DN36395_c0_g1_i1_4, TRINITY_DN29693_c0_g1_i1_4, TRINITY_DN31947_c0_g1_i1_4, TRINITY_DN29512_c0_g1_i1_4, TRINITY_DN26616_c0_g1_i1_4, TRINITY_DN26835_c0_g1_i1_4
03051 Proteasome	A09180 Brite Hierarchies	9	0.01083547 1	2.445128758	TRINITY_DN8568_c0_g1_i1_4, TRINITY_DN33313_c0_g1_i1_4, TRINITY_DN28880_c0_g1_i1_4, TRINITY_DN36395_c0_g1_i1_4, TRINITY_DN29693_c0_g1_i1_4, TRINITY_DN31947_c0_g1_i1_4, TRINITY_DN29512_c0_g1_i1_4, TRINITY_DN26616_c0_g1_i1_4, TRINITY_DN26835_c0_g1_i1_4
04121 Ubiquitin system	A09180 Brite Hierarchies	9	0.01083547 1	2.445128758	TRINITY_DN8568_c0_g1_i1_4, TRINITY_DN33313_c0_g1_i1_4, TRINITY_DN28880_c0_g1_i1_4, TRINITY_DN36395_c0_g1_i1_4, TRINITY_DN29693_c0_g1_i1_4, TRINITY_DN31947_c0_g1_i1_4, TRIN-

					ITY_DN29512_c0_g1_i1_4, TRIN-ITY_DN26616_c0_g1_i1_4, TRIN-ITY_DN26835_c0_g1_i1_4
03050 Proteasome	A09120 Genetic Information Pro-cessing	9	0.010835471	2.445128758	TRINITY_DN8568_c0_g1_i1_4, TRIN-ITY_DN33313_c0_g1_i1_4, TRIN-ITY_DN28880_c0_g1_i1_4, TRIN-ITY_DN36395_c0_g1_i1_4, TRIN-ITY_DN29693_c0_g1_i1_4, TRIN-ITY_DN31947_c0_g1_i1_4, TRIN-ITY_DN29512_c0_g1_i1_4, TRIN-ITY_DN26616_c0_g1_i1_4, TRIN-ITY_DN26835_c0_g1_i1_4
B 09103 Lipid me-tabolism	A09100 Metabo-lism	28	0.017124862	1.517756205	TRINITY_DN31687_c0_g1_i1_4, TRINITY_DN127_c0_g2_i1_6, TRIN-ITY_DN3062_c0_g2_i1_4, TRIN-ITY_DN31880_c0_g1_i1_4, TRIN-ITY_DN33884_c0_g1_i1_4, TRIN-ITY_DN30746_c0_g1_i1_4, TRIN-ITY_DN29435_c0_g1_i1_6, TRIN-ITY_DN30161_c0_g1_i1_6, TRIN-ITY_DN3293_c0_g1_i1_4, TRIN-ITY_DN37346_c0_g1_i1_4, TRIN-ITY_DN27451_c0_g1_i1_4, TRIN-ITY_DN27394_c0_g1_i1_4, TRIN-ITY_DN38780_c0_g1_i1_6, TRIN-ITY_DN31588_c0_g1_i1_4, TRIN-ITY_DN35640_c0_g1_i1_4, TRIN-ITY_DN28649_c0_g1_i1_4, TRIN-ITY_DN26605_c0_g1_i1_4, TRIN-ITY_DN31949_c0_g1_i1_4, TRIN-ITY_DN29834_c0_g1_i1_6, TRIN-ITY_DN29924_c0_g1_i1_4, TRIN-ITY_DN35533_c0_g1_i1_4, TRIN-ITY_DN35824_c0_g1_i1_4, TRIN-ITY_DN29940_c0_g1_i1_4, TRIN-

					ITY_DN28926_c0_g1_i1_4, TRINITY_DN33593_c0_g1_i1_4, TRINITY_DN4679_c0_g1_i1_6, TRINITY_DN27308_c0_g1_i1_4, TRINITY_DN37350_c0_g1_i1_4

Table S2. Graphs present 14 KEGG pathways with the highest transcriptional variations, out of the up-regulated DEGs in *Paspalum vaginatum.*

Term Name	MainClass	GeneHitsInSelectedSet	p-value	enrichFactor	GeneListInSelectedSets
03016 Transfer RNA biogenesis	A09180 Brite Hierarchies	15	8.26E-06	0.41204	TRINITY_DN17249_c0_g1_i1_2, TRINITY_DN26978_c0_g1_i1_1, TRINITY_DN10409_c0_g1_i4_1, TRINITY_DN7420_c0_g1_i1_2, TRINITY_DN18386_c0_g1_i4_2, TRINITY_DN25122_c0_g1_i8_2, TRINITY_DN8697_c0_g1_i1_2, TRINITY_DN23660_c0_g2_i1_2, TRINITY_DN24792_c0_g1_i18_2, TRINITY_DN21259_c0_g1_i6_5, TRINITY_DN13877_c0_g1_i1_2, TRINITY_DN8539_c0_g1_i1_3, TRINITY_DN11896_c0_g1_i4_2, TRINITY_DN17375_c0_g1_i1_3, TRINITY_DN19525_c1_g1_i1_1
00660 C5-Branched dibasic acid metabolism	A09100 Metabolism	9	2.89E-05	0.43110	TRINITY_DN22555_c0_g2_i7_3, TRINITY_DN23869_c1_g1_i9_2, TRINITY_DN15585_c0_g1_i2_6, TRINITY_DN9193_c0_g1_i1_6, TRINITY_DN22470_c0_g1_i4_2, TRINITY_DN17831_c0_g1_i1_3, TRINITY_DN24324_c0_g1_i1_5, TRINITY_DN21552_c0_g1_i2_3, TRINITY_DN21482_c0_g1_i3_3

00290 Valine, leucine and isoleucine biosynthesis	A09100 Metabolism	11	7.36E-05	0.431108	TRINITY_DN22555_c0_g2_i7_3, TRINITY_DN23869_c1_g1_i9_2, TRINITY_DN15585_c0_g1_i2_6, TRINITY_DN9193_c0_g1_i1_6, TRINITY_DN22470_c0_g1_i4_2, TRINITY_DN17831_c0_g1_i1_3, TRINITY_DN24061_c0_g1_i1_2, TRINITY_DN25983_c0_g3_i1_5, TRINITY_DN24324_c0_g1_i1_5, TRINITY_DN21552_c0_g1_i2_3, TRINITY_DN21482_c0_g1_i3_3
B 09105 Amino acid metabolism	A09100 Metabolism	22	0.007248	0.431108	TRINITY_DN21352_c0_g1_i32_1, TRINITY_DN23869_c1_g1_i9_2, TRINITY_DN15585_c0_g1_i2_6, TRINITY_DN22470_c0_g1_i4_2, TRINITY_DN9193_c0_g1_i1_6, TRINITY_DN21326_c0_g1_i17_3, TRINITY_DN17831_c0_g1_i1_3, TRINITY_DN21720_c0_g1_i8_3, TRINITY_DN25983_c0_g3_i1_5, TRINITY_DN24324_c0_g1_i1_5, TRINITY_DN21482_c0_g1_i3_3, TRINITY_DN22555_c0_g2_i7_3, TRINITY_DN18616_c0_g1_i9_2, TRINITY_DN23809_c0_g4_i8_3, TRINITY_DN25467_c0_g1_i10_2, TRINITY_DN25579_c0_g1_i7_6, TRINITY_DN24061_c0_g1_i1_2, TRINITY_DN25240_c0_g1_i14_2, TRINITY_DN22255_c0_g2_i20_1, TRINITY_DN21552_c0_g1_i2_3, TRINITY_DN23285_c0_g1_i10_2, TRINITY_DN24132_c0_g2_i11_2
B 09101 Carbohydrate metabolism	A09100 Metabolism	19	0.006482	0.423887	TRINITY_DN21352_c0_g1_i32_1, TRINITY_DN23869_c1_g1_i9_2, TRINITY_DN15585_c0_g1_i2_6,

					TRINITY_DN16544_c0_g1_i2_3, TRINITY_DN24039_c1_g1_i16_2, TRINITY_DN22470_c0_g1_i4_2, TRINITY_DN9193_c0_g1_i1_6, TRINITY_DN21326_c0_g1_i17_3, TRINITY_DN17831_c0_g1_i1_3, TRINITY_DN23953_c0_g1_i19_2, TRINITY_DN24324_c0_g1_i1_5, TRINITY_DN21482_c0_g1_i3_3, TRINITY_DN22555_c0_g2_i7_3, TRINITY_DN25042_c0_g2_i2_2, TRINITY_DN17068_c0_g1_i5_1, TRINITY_DN21552_c0_g1_i2_3, TRINITY_DN23285_c0_g1_i10_2, TRINITY_DN19877_c0_g1_i1_1, TRINITY_DN24132_c0_g2_i11_2
B 09108 Metabolism of cofactors and vitamins	A09100 Metabolism	14	0.005467	0.744515	TRINITY_DN23869_c1_g1_i9_2, TRINITY_DN24722_c0_g1_i24_2, TRINITY_DN22470_c0_g1_i4_2, TRINITY_DN21326_c0_g1_i17_3, TRINITY_DN25983_c0_g3_i1_5, TRINITY_DN21482_c0_g1_i3_3, TRINITY_DN22555_c0_g2_i7_3, TRINITY_DN23809_c0_g4_i8_3, TRINITY_DN20232_c0_g1_i3_4, TRINITY_DN25579_c0_g1_i7_6, TRINITY_DN24061_c0_g1_i1_2, TRINITY_DN20392_c0_g1_i3_4, TRINITY_DN22255_c0_g2_i20_1, TRINITY_DN23285_c0_g1_i10_2
A09100 Metabolism	A09100 Metabolism	41	0.017723	0.795616	TRINITY_DN21352_c0_g1_i32_1, TRINITY_DN24039_c1_g1_i16_2, TRINITY_DN21326_c0_g1_i17_3, TRINITY_DN17831_c0_g1_i1_3, TRINITY_DN21720_c0_g1_i8_3, TRINITY_DN23953_c0_g1_i19_2,

					TRINITY_DN24198_c0_g4_i1_2, TRINITY_DN21482_c0_g1_i3_3, TRINITY_DN16223_c0_g1_i8_1, TRINITY_DN22555_c0_g2_i7_3, TRINITY_DN25042_c0_g2_i2_2, TRINITY_DN23809_c0_g4_i8_3, TRINITY_DN20232_c0_g1_i3_4, TRINITY_DN24104_c7_g1_i3_3, TRINITY_DN24061_c0_g1_i1_2, TRINITY_DN23285_c0_g1_i10_2, TRINITY_DN24082_c1_g2_i15_3, TRINITY_DN24132_c0_g2_i11_2, TRINITY_DN23869_c1_g1_i9_2, TRINITY_DN15585_c0_g1_i2_6, TRINITY_DN16544_c0_g1_i2_3, TRINITY_DN22934_c0_g1_i2_2, TRINITY_DN8538_c0_g1_i2_2, TRINITY_DN23416_c0_g1_i9_3, TRINITY_DN24722_c0_g1_i24_2, TRINITY_DN22470_c0_g1_i4_2, TRINITY_DN9193_c0_g1_i1_6, TRINITY_DN21684_c0_g1_i3_3, TRINITY_DN25983_c0_g3_i1_5, TRINITY_DN24324_c0_g1_i1_5, TRINITY_DN19096_c0_g4_i1_1, TRINITY_DN18616_c0_g1_i9_2, TRINITY_DN17068_c0_g1_i5_1, TRINITY_DN25467_c0_g1_i10_2, TRINITY_DN25579_c0_g1_i7_6, TRINITY_DN25240_c0_g1_i14_2, TRINITY_DN20392_c0_g1_i3_4, TRINITY_DN22709_c0_g1_i6_3, TRINITY_DN22255_c0_g2_i20_1, TRINITY_DN21552_c0_g1_i2_3, TRINITY_DN19877_c0_g1_i1_1
00130 Ubiquinone and other terpenoid-quinone biosynthesis	A09100 Metabolism	2	0.032161	0.243044	TRINITY_DN24722_c0_g1_i24_2, TRINITY_DN20232_c0_g1_i3_4

00620 Pyruvate metabolism	A09100 Metabolism	5	0.027258	0.305277	TRINITY_DN23869_c1_g1_i9_2, TRINITY_DN16544_c0_g1_i2_3, TRINITY_DN17068_c0_g1_i5_1, TRINITY_DN22470_c0_g1_i4_2, TRINITY_DN21482_c0_g1_i3_3
00710 Carbon fixation in photosynthetic organisms	A09100 Metabolism	5	0.027258	0.637771	TRINITY_DN23869_c1_g1_i9_2, TRINITY_DN16544_c0_g1_i2_3, TRINITY_DN17068_c0_g1_i5_1, TRINITY_DN22470_c0_g1_i4_2, TRINITY_DN21482_c0_g1_i3_3
B 09106 Metabolism of other amino acids	A09100 Metabolism	3	0.079691	0.632437	TRINITY_DN24039_c1_g1_i16_2, TRINITY_DN21326_c0_g1_i17_3, TRINITY_DN23285_c0_g1_i10_2
00790 Folate biosynthesis	A09100 Metabolism	7	0.04543	0.741387	TRINITY_DN22555_c0_g2_i7_3, TRINITY_DN23869_c1_g1_i9_2, TRINITY_DN23809_c0_g4_i8_3, TRINITY_DN22470_c0_g1_i4_2, TRINITY_DN25579_c0_g1_i7_6, TRINITY_DN22255_c0_g2_i20_1, TRINITY_DN21482_c0_g1_i3_3
00360 Phenylalanine metabolism	A09100 Metabolism	7	0.04543	0.75406	TRINITY_DN22555_c0_g2_i7_3, TRINITY_DN23869_c1_g1_i9_2, TRINITY_DN23809_c0_g4_i8_3, TRINITY_DN22470_c0_g1_i4_2, TRINITY_DN25579_c0_g1_i7_6, TRINITY_DN22255_c0_g2_i20_1, TRINITY_DN21482_c0_g1_i3_3
00400 Phenylalanine, tyrosine and tryptophan biosynthesis	A09100 Metabolism	7	0.04543	0.770524	TRINITY_DN22555_c0_g2_i7_3, TRINITY_DN23869_c1_g1_i9_2, TRINITY_DN23809_c0_g4_i8_3, TRINITY_DN22470_c0_g1_i4_2, TRINITY_DN25579_c0_g1_i7_6,

					TRINITY_DN22255_c0_g2_i20_1, TRINITY_DN21482_c0_g1_i3_3
00600 Sphingolipid metabolism	A09100 Metabolism	3	0.043074	0.79482	TRINITY_DN8538_c0_g1_i2_2, TRINITY_DN23416_c0_g1_i9_3, TRINITY_DN21684_c0_g1_i3_3
